# A low voltage nanopipette dielectrophoretic device for rapid entrapment of nanoparticles and exosomes extracted from plasma of healthy donors

**DOI:** 10.1038/s41598-018-25026-2

**Published:** 2018-04-30

**Authors:** Leilei Shi, Ankit Rana, Leyla Esfandiari

**Affiliations:** 1 Department of Electrical Engineering and Computer Science, College of Engineering and Applied Science, University of Cincinnati, Ohio, 45221 United States; 2 Department of Biomedical Engineering, College of Engineering and Applied Science, University of Cincinnati, Ohio, 45221 United States

## Abstract

An insulator-based dielectrophoresis (iDEP) is a label-free method that has been extensively utilized for manipulation of nanoparticles, cells, and biomolecules. Here, we present a new iDEP approach that can rapidly trap nanoparticles at the close proximity of a glass nanopipette’s tip by applying 10 V/cm direct current (DC) across the pipette’s length. The trapping mechanism was systemically studied using both numerical modeling and experimental observations. The results showed that the particle trapping was determined to be controlled by three dominant electrokinetic forces including dielectrophoretic, electrophoretic and electroosmotic force. Furthermore, the effect of the ionic strength, the pipette’s geometry, and the applied electric field on the entrapment efficiency was investigated. To show the application of our device in biomedical sciences, we demonstrated the successful entrapment of fluorescently tagged liposomes and unlabeled plasma-driven exosomes from the PBS solution. Also, to illustrate the selective entrapment capability of our device, 100 nm liposomes were extracted from the PBS solution containing 500 nm polystyrene particles at the tip of the pipette as the voltage polarity was reversed.

## Introduction

In the last few decades, considerable efforts have been made to develop new miniaturized technologies for particle manipulation, pre-concentration, and sorting using the optical^1–3^, magnetic^4–6^, acoustic^7–9^, and dielectrophoretic (DEP)^10–18^ schemes. Among these technologies, the DEP principle has gained significant attention because of its rapid and truly label-free criteria^19^. DEP has been utilized for sorting^10–12^, isolating^13,14^, and manipulating^15,16^ of micro/nano-scaled particles and biomolecules, based on their dielectric properties under the non-uniform electric field (E-field)^18,20,21^. The non-uniformity of the E-field can be generated either by applying an alternating current (AC) across an array of electrodes^10,22–30^ or by placing obstacles such as micro-pillars and rectangular hurdles in microfluidic channels (insulator-based approach)^17,31,32^. The “insulator-based” (iDEP) approach is more appealing for manipulation of biomolecules because the device functionality can be preserved despite the fouling effect at the electrodes’ surface^18,33^. Although this approach has shown promising attributes, the majority of iDEP devices require high operational voltage in the range of 100 V/cm^11,32,34,35^ which could result in generation of excessive heat in the system and potentially denaturation of the biological entitles.

An alternative iDEP approach was initially introduced by the Klenerman group^36^, as significantly lower voltage was applied across a glass nanopipette; The small conical geometry of the nanopore induced a strong non-uniform E-field which created a DEP trapping zone inside of the pipette near the tip region^36–38^. This method has been used for trapping DNA molecules and proteins, by backfilling the nanopipette with a solution containing the target analytes and concentrating them inside the pipette as an AC field was applied^36–39^. Although this technique addressed the need for low operational voltage, it is unable to directly trap analytes from the bulk solution. To address this, Heller’s team has developed a pipette based DEP device with a larger inner diameter of 800 μm which could directly isolate microbeads and DNA molecules from the bulk solution^40^. In a similar strategy, a metal coated glass nanopipette was utilized to collect biomolecules under an AC field^41,42^. Furthermore, in another clever method, a pressure gradient was initially applied to drive DNA molecules close to the tip of a glass pipette and then the balance of the electroosmotic flow (EOF), the pressure-driven flow, and the electrophoresis (EP) was used to trap them^43^.

The majority of the nanopipette-DEP schemes are focused on the entrapment of analytes under the AC field and a comprehensive analysis of trapping mechanism under the DC has not yet been reported. In this work, we demonstrated a nanopipette-DEP device that is capable of rapid entrapment of nanoparticles at the close proximity region in front of the pipette’s tip, under the low applied DC field, from solution with various ionic strengths. As a model system, 510 nm carboxylic acid polystyrene (COOH-PS) beads have been used to comprehensively study the system’s electrokinetic (EK) forces including EP, DEP and EOF. The correlation between the induced EK forces and the number of trapped particles was systematically investigated by numerical modeling and experimental observations as the physical parameters, such as the applied voltage, solution ionic strength, and the pipette diameters were varied. Furthermore, to demonstrate the capability of our device for selective entrapment of nano-vesicles based on their dielectric properties and size, fluorescently tagged artificial liposomes with 100 nm diameters were selectively sorted and pre-concentrated from the PBS solution containing 510 nm COOH-PS beads. Also, to show the biomedical application of our device, small extracellular vesicles (exosomes) extracted from plasma of healthy donors, re-suspended in PBS solution, were trapped and pre-concentrated under two minutes.

## Results and Discussion

### The effect of magnitude and polarity of applied potential on particles entrapment

**Figure 1:**
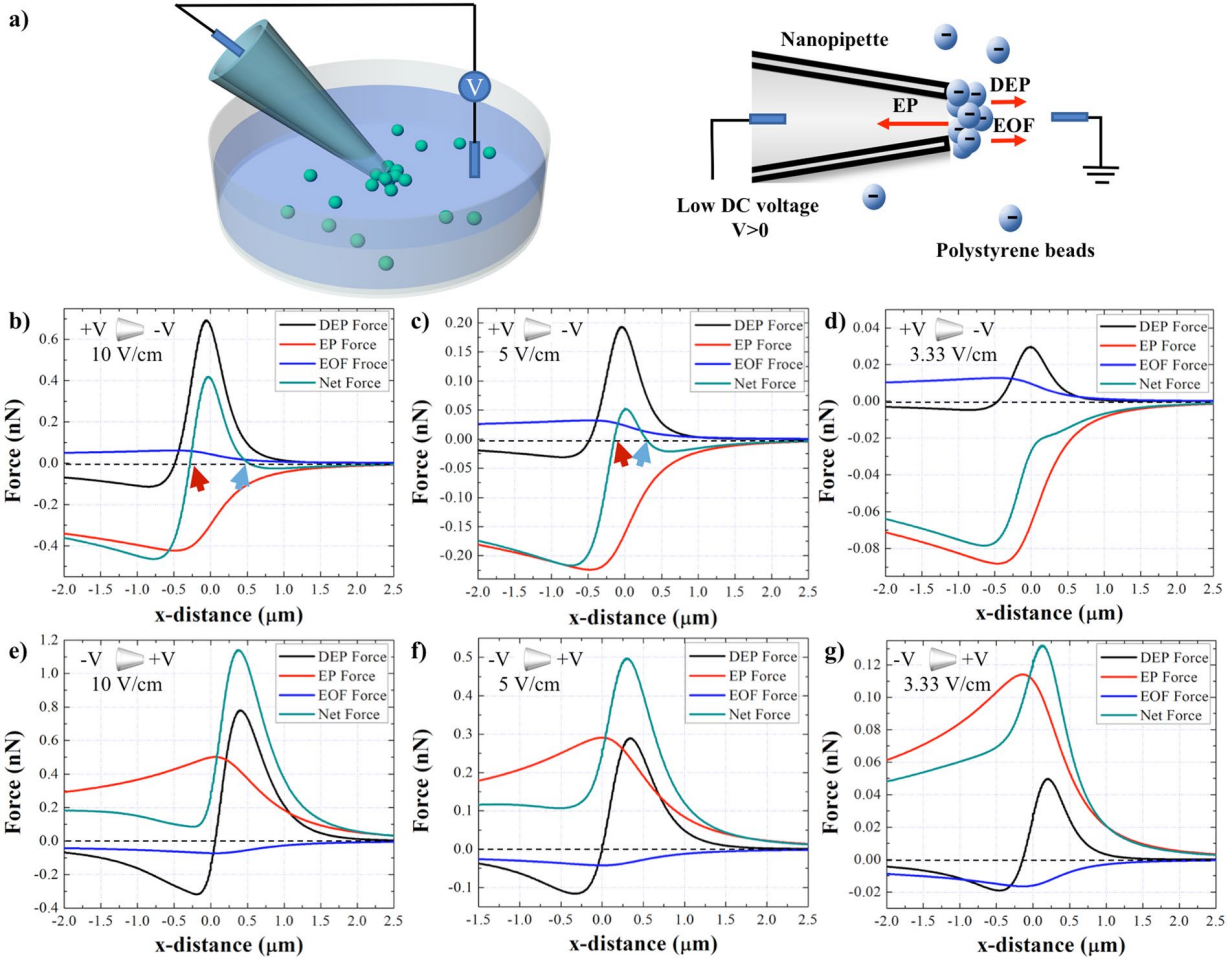
(**a**) The schematic of the nanopipette DEP device for entrapment of the particles suspended in the solution. (**b**–**d**) DEP, EP, EOF, and the net EK forces induced on the 510 nm COOH-PS beads along the length of the pipette under positive bias of 10, 5, and 3.33 V/cm applied at the base, respectively. (**e**–**g**) DEP, EP, EOF, and the net EK forces induced on the 510 nm COOH-PS beads along the length of the pipette under negative bias of 10, 5, and 3.33 V/cm applied at the base, respectively. The part of the graph to the left of (0,0) represents the region inside of the pipette and the portion of the graph on the right of (0, 0) depicts the region outside the pipette. The red and blue arrows point out the intersection between the net EK force plot and Y=0. The ionic strength of solution is 10 mM and the diameter of the pore is 1 μm.

To realize the physical principles of the system, a finite element simulation was carried out to study the EK forces induced on COOH-PS beads using the nanopipette-DEP device with a 1 μm inner diameter in monovalent ionic condition at 10 mM concentration. Figure 1a illustrates the concept of the device in which as the direct current (DC) was applied across the pipette, the suspended particles in the solution were conveyed to the region close to the pipette’s tip and were trapped near the tip region due to the balance of three electrokinetic forces at that region. Figure 1b–g shows the EK forces, including the EP, EOF, DEP, and the net force induced on the particles along the central axis of the pipette under different magnitudes and polarities of DC potential. As a positive voltage bias was applied at the base of the pipette (Fig. 1b–d), the distribution of EP force shows a peak in the negative Y-axis which indicates its direction towards the pipette’s tip. However, the EOF and DEP forces had peaks in the positive Y-axis and thus, their directions were away from the pipette. Furthermore, under the 10 and 5 V/cm, the net force crossed the X-axis at two regions: inside of the piptette (red arrow), and outside of the piptte (blue arrow) (Fig. 1b,c) which represents the force balance at those two regions and hence, the trapping zones. However, as the applied voltage decreased to 3.33 V/cm, the net force did not cross the X-axis (Fig. 1d), since the combination of DEP and EOF forces was not sufficient to counter balance the EP force and thus, the particles were expected to translocate in to the pipette.

In another set of simulations, the polarity of the voltage was reversed and the negative bias was applied at the base of the pipette (Fig. 1e–g). The EOF showed a peak in the negative Y-axis, implying that its orientation was toward the base of the pipette. Meanwhile, the DEP force continued to have an emanating effect as E-field gradient is not affected by the voltage polarity. However, the EP force direction switched as the voltage polarity was reversed and showed a peak at the positive Y-axis which implies its outward orientation away from the tip. As a result, the direction of the cumulative force was outward and hence, pushed the particles away from the tip. Thus, the particles were expected to not be trapped with reversal of the voltage polarity (Fig. 1e–g).

**Figure 2:**
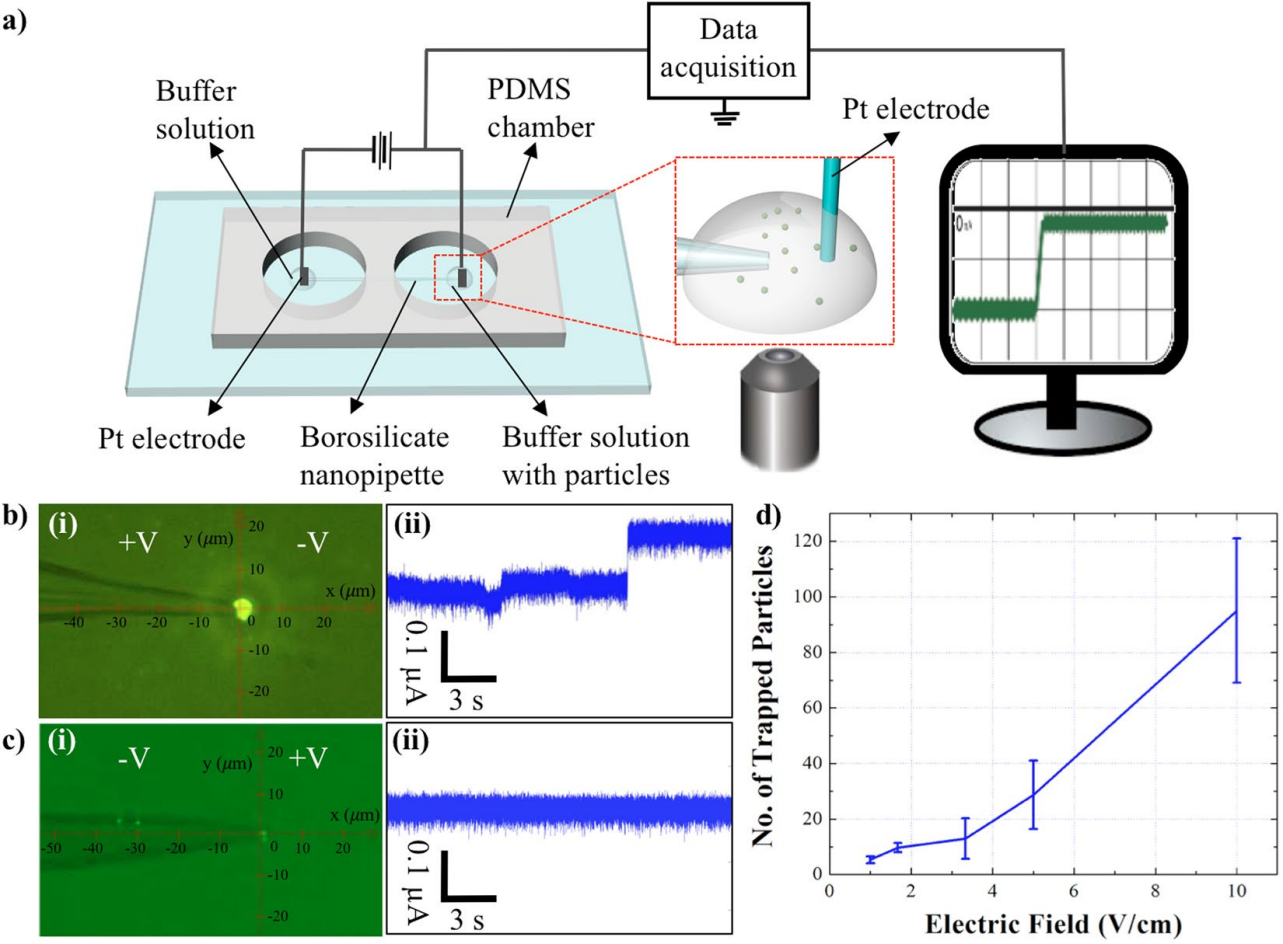
(**a**) A schematic of the nanopipette-DEP device. The ionic current across the pipette and the trajectory of the particles were simultaneously recorded. (**b**) (i) Microscopic image of entrapment as 10 V/cm was applied with the positive bias at the base of the pipette after 100 seconds. (**b**) (ii) The conductance measurements across the 1 μm pore as the particles accumulate by the tip. (**c**) (i) Microscopic image of few particles entrapment as negative 10 V/cm bias was applied at the base of the pipette after 100 seconds. (**c**) (ii) The corresponding conductance measurements across the 1 μm pore. (**d**) The correlation between the magnitude of electric field and the number of trapped particles after 100 seconds. The suspending medium was 10 mM KCl (pH = 7.0).

The simulations were followed by experiments in which 510 nm fluorescently-tagged (COOH-PS) beads suspended in 10 mM KCl (pH 7.0) were injected in the chamber facing a 1 μm pipette. 10 V/cm bias was applied across the pipette while the the motion of the particles was microscopically recorded and the ionic current across the pore was measured (Fig. 2a). Figure 2b illustrates the entrapment of the particles after 100 seconds of applied positive potential at the base of the pipette. The conductance measurement across the pipette shows ionic current step changes as the particles accumulated by the tip (Fig. 2b(ii)). The conductance measurements could potentially be translatable as a qualitative indicator for particles entrapment in the cases where the optical resolution is limited. However, no significant entrapment was observed when the same experiment was repeated with the reversed voltage polarity (Fig. 2c) and the open pore current (Fig. 2c(ii)) with no step changes was obtained (Video S1 Supporting Information). Furthermore, consistent results were obtained when negative bias was initially applied at the base followed by the reversal of the voltage polarity (data not shown).

It has also been observed that the trapped beads could not be completely released from the trapping zone as the voltage polartiy was reversed and thus, the experiments could not be repeated using the same nanopipette under the solution with 10 mM ionic strength. However, as the solution’s ionic strenght decreased significantly, for instantce, in the case of the beads suspended in DI water, the particles could be removed from the trapping zone as the voltage polarity was reveresed because of the strong opposing EOF force (data not shown)^44^. Thus, under the very low ionic strength condition, the same nanopipette could potenitally be recycled and re-used in various experiments.

In another set of experiments the magnitude of voltage was systematically reduced from 10 to 5, 3.33, 1.67, 1, and 0.67 V/cm to establish the minimum voltage required for particles entrapment. Figures 2d and S1 show approximate number of trapped particles under different voltage magnitudes which indicate the entrapment of a few particles under 1 V/cm and more significant entrapment as 5 V/cm was applied.

The experimental results contradicted the simulations only in the case where no particle was translocated through the pore as 3.33 V/cm positive bias was applied at the base. A possible explanation could be the negligence of the electrostatic repulsion between the beads and the pipette’s surface in the numerical analysis.

### The effect of solution ionic strength on particles entrapment

**Figure 3:**
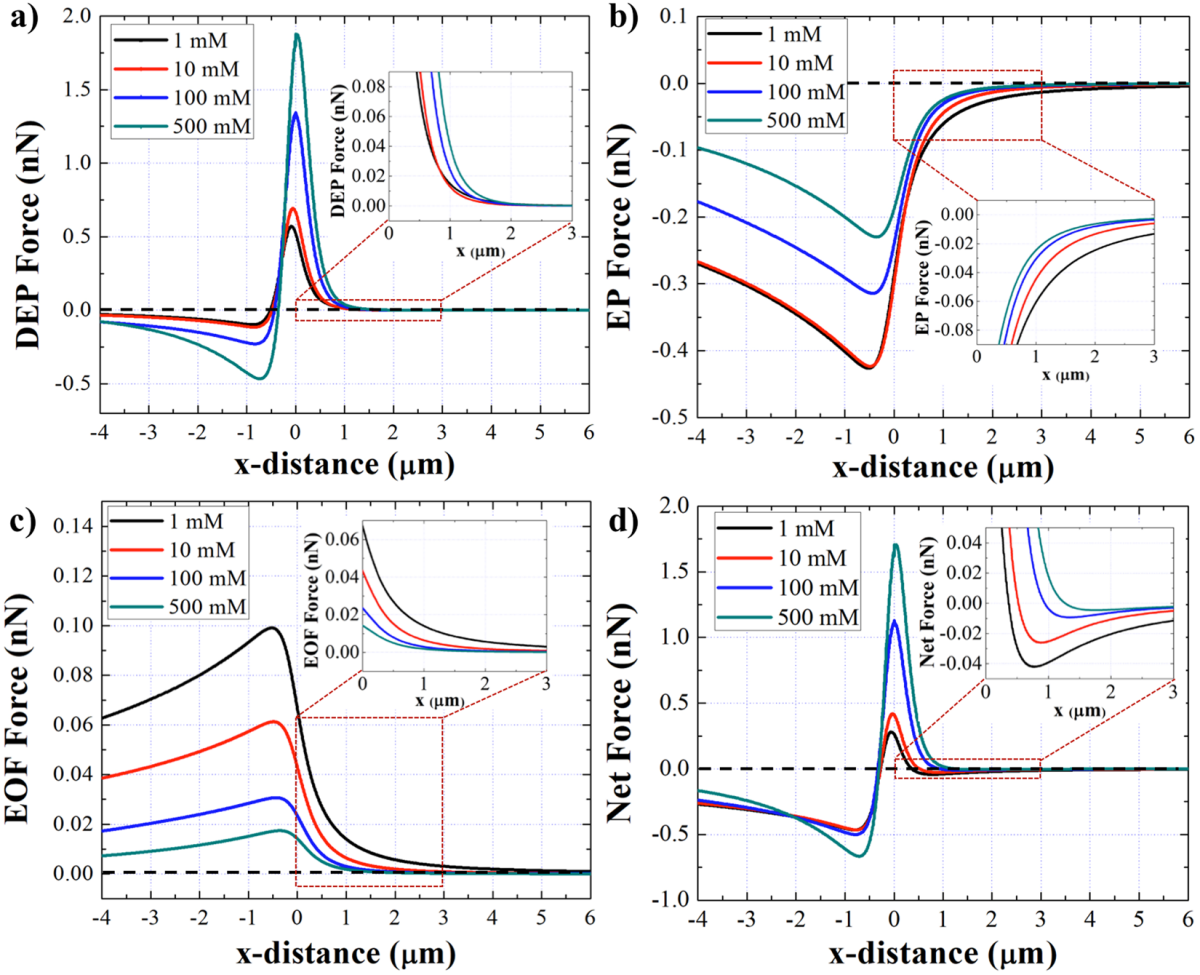
(**a**–**d**) The plot of DEP, EP, EOF and the net force applied on the 510 nm COOH-PS beads along the length of the 1 μm pipette under different ionic strengths. A positive 10 V/cm bias was applied at the base of the pipette. The zoom in figures show the distribution of EK forces within a close distance from the pipette’s tip at the trapping zone.

The performance of the device was investigated numerically and experimentally under solution with different ionic strengths. Simulation results represent the magnitude and direction of the EK forces induced on the COOH-PS beads suspended in various salt concentrations using the 1 μm pipette, under the 10 V/cm positive bias applied at the pipette’s base (Fig. 3). To better illustrate the distribution of the forces at the trapping zone, the zoom in image of each EK force in front of the pipette where the force balance occurs, was plotted. Figure 3a shows the DEP force distribution along the length of the pipette. The direction of the induced DEP force on the particles in all ionic strength conditions was negative (nDEP) since the conductivity of the solution was higher than the conductivity of the particles. Although the direction of the DEP force remained the same, the magnitude of the DEP increased as the salt concentration increased. Moreover, the magnitude of the EP force applied on the particles was reduced as the solution ionic strength increased due to the decrease of the Debye length (Fig. 3b). A similar trend was observed in the case of the EOF profile due to decrease of the Debye length close to the Silanol groups on the pipette’s wall (Fig. 3c). Figure 3d represents the cumulative EK forces computed along the length of the pipette at various ionic strengths and the zoom in image illustrates the reduction of the net force at the trapping zone as the solution’s salt concentration increased.

**Figure 4:**
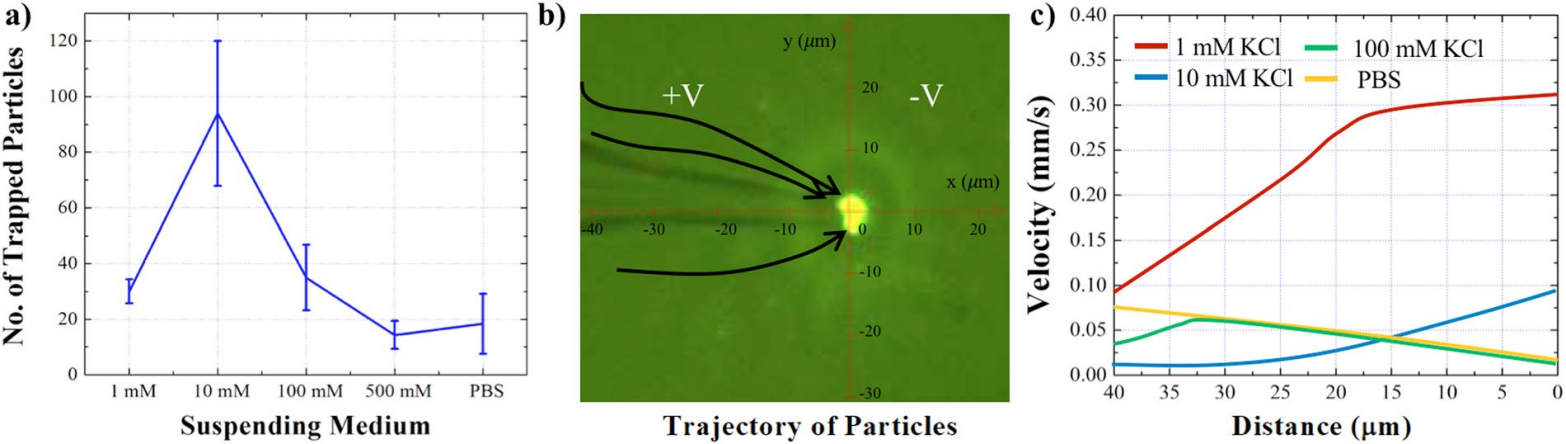
(**a**) The correlation between the estimated number of trapped particles and the ionic strength of the solution at pH 7.0. Each experiment was conducted using a 1 μm pipette under 10 V/cm and the data was collected at 100 seconds after the positive voltage was applied at the base of the pipette. (**b**) The trajectory (black arrows) of the beads motion before getting trapped at the tip, (0, 0) point. (**c**) The velocity of the COOH-PS beads suspended in solutions with different salt concentrations with respect to their distance from the tip, point (0, 0). The distance was obtained from the particles’ coordinate with respect to the pipette’s tip as shown in figure b).

Experiments under the same conditions were conducted and the approximate number of entrapped particles was quantified after 100 seconds of applied potential (Figs 4a, S3). Although the simulation results show a reversed linear regression of the net EK force with respect to the solution’s salt concentration, the experimental results did not follow the same trend for the 1 mM KCl condition (Fig. 4a). This puts forth the hypothesis that the trapping mechanism is not only governed by the forces acting on the particles, but also by the particles’ initial position with respect to the pipette prior to entering the trapping zone. In the area far away from the tip, the electric field and the electric field gradient are insignificant and thus, the EP and DEP forces are negligible. However, the electroosmotic flow exists near the pipette’s outer walls even within a far distance from the tip. Therefore, particles close to the pipette’s walls could be influenced by the EOF and obtain an initial velocity before entering the trapping zone compared to the particles positioned in the bulk that are away from the pipette’s outer wall. To confirm this, the EOF distribution along the pipettes’ outer walls was simulated at various distances from the walls (Fig. S3) and as expected, the results show a decrease in the induced EOF on the particles located further away from the walls. Moreover, the EOF distribution along the pipettes’ walls was simulated in solution with different ionic strengths (Fig. S4) which indicates the reduction of the EOF as the ionic strength increased. Thus, we postulate that particles suspended in a low ionic strength solution, i.e. 1 mM KCl, have higher average velocity prior to entering the trapping zone and as a result the net EK force in front of the pipette’s tip was not strong enough to halt the particles at the tip (trapping zone) and hence, led to fewer particles entrapment. To further support our argument, motion of the particles was tracked using the microscopic footage (Fig. 4b) and the results indicate a significantly higher particles’ velocity when particles were suspended in solutions with lower salt concentration due to the higher EOF on the outer walls of the pipette (Fig. 4c). Also, in the case of the higher ionic strength conditions, i.e. 100 mM KCl, the velocity of the particles decreased gradually and approached almost zero as they were reaching the trapping zone. The low velocity of the particles could potentially result in lower entrapment yield since the majority of the particles did not have sufficient speed to reach the trapping zone. However, by analyzing the particles’ motion suspended in 10 mM KCl, we observed that the particles’ velocity increased gradually and attained approximately 0.1 mm/sec at the tip of the pipette (the trapping zone). By comparing the velocity of the particles in 10, 100, and 1 mM KCl we observed that the velocity of the particles suspended in 10 mM ionic strength was higher than the particles’ velocity suspended in 100 mM KCl solution, yet lower than the particles’ velocity suspended in 1 mM KCl solution. Thus, the velocity of the particles in 10 mM ionic strength was sufficient enough to reach the trapping zone and moderate enough to be halted by the net EK trapping force in front of the pipette. This intermediate particles’ velocity under the 10 mM ionic strength condition, increased the trapping yield while compared to the particles’ velocity suspened in the other solutions.

### The effect of the nanopipette’s geometry on particles entrapment

**Figure 5:**
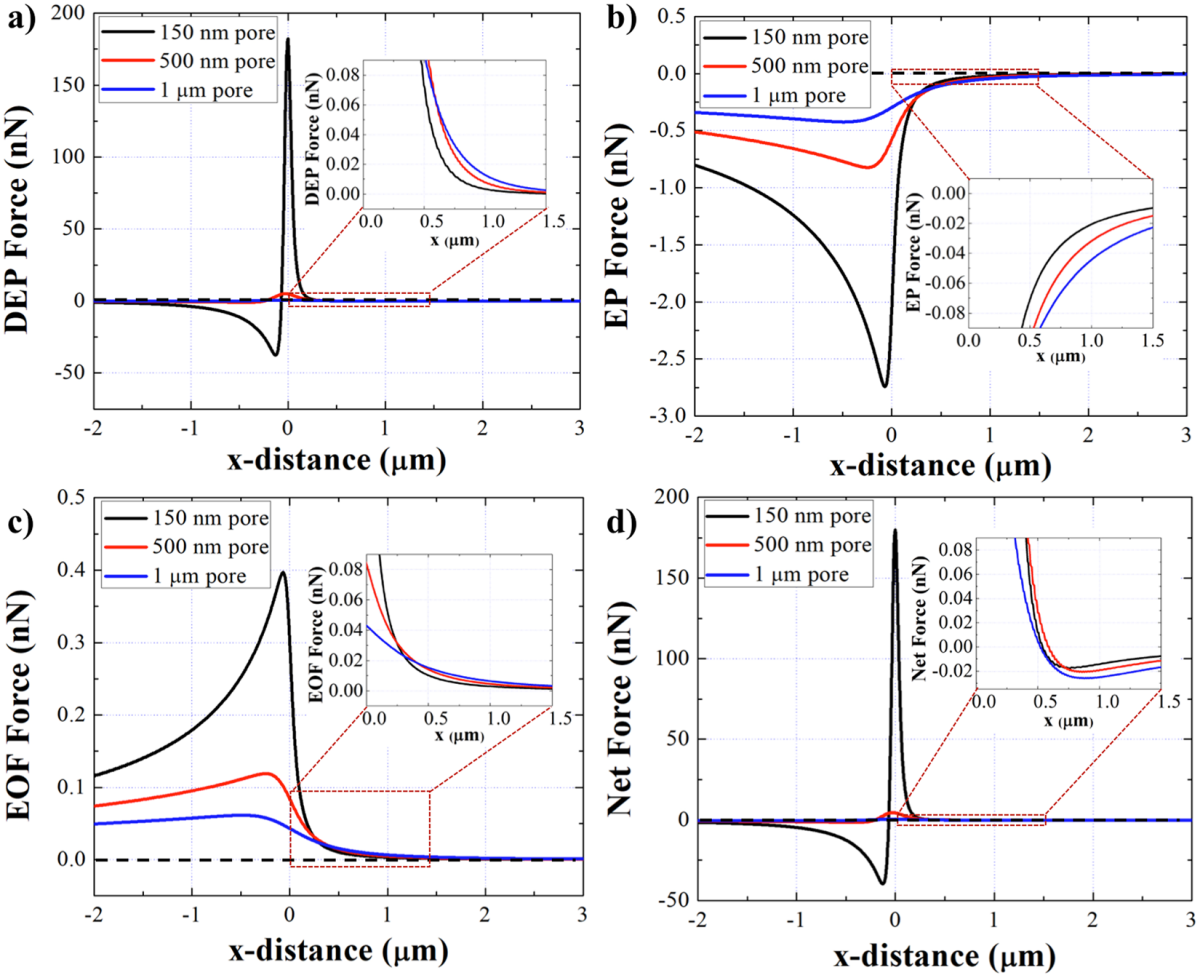
The distribution of EK forces applied on the 510 nm COOH-PS beads in 10 mM KCl with different pore diameters under positve 10 V/cm bias at the base. (**a**) DEP, (**b**) EP, (**c**) EOF and (**d**) the net EK forces along the length of the pipette. The zoom in inserted figures show the distribution of forces in fornt of the pipette’s tip at the trapping zone where the forces are balanced.

The effect of the pipette’s diameter on entrapment was numerically investigated with pores of 150 nm, 500 nm and 1 μm diameters. Figure 5 shows the distribution of the DEP, EP, EOF and the net EK forces along the length of the pipettes with three different pore sizes. The results show, at the trapping zone, near the region in front of the tip where the force balance occurs (Fig. 5a,b inserts), the DEP and EP forces drop faster as the pore size decreased. In addition, at the trapping zone, the electroosmosis flow drops faster as the pore diameter decreased due to the higher resistance of the smaller pores (Fig. 5c insert). Consequently, the net EK force distribution at the trapping zone, illustrates an increase in the magnitude of the net force as the pipettes’ diameter increased which is expected to lead to greater number of particles entrapment.

**Figure 6:**
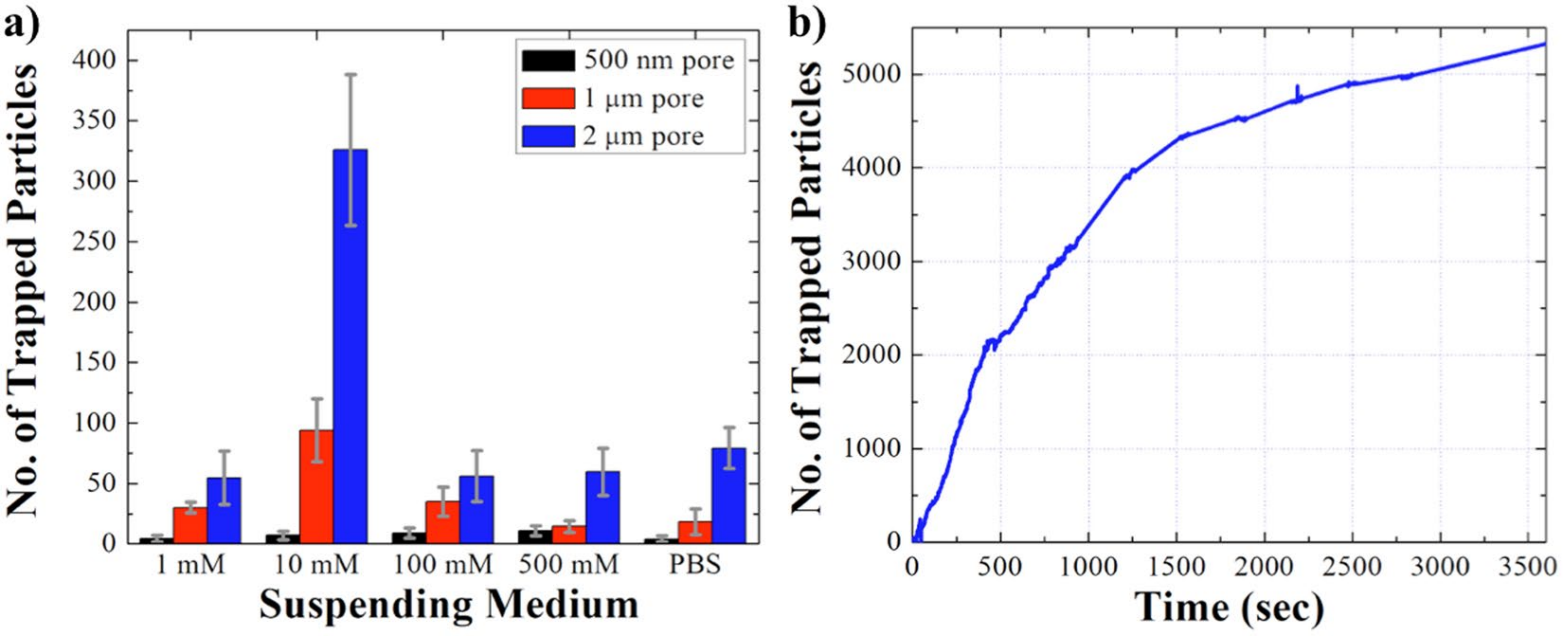
(**a**) The correlation between the number of trapped particles, the ionic strength of the solution, and the pipettes’ diameter. Each experiment was conducted under 10 V/cm with positive bias applied at the base of the pipette and the data was collected after 100 seconds of applied voltage. (**b**) The correlation between the number of trapped particles and the run time. The COOH-PS beads were suspended in 10 mM KCl pH 7.0, and 10 V/cm was applied at the base of the 2 μm pipette.

Emperical observations using the 500 nm, 1 μm, and 2 μm pipettes illustrated the entrapment yeild similar to the simulation results (Fig. 6a). Also, the effect of the solution’s ionic strength was investigated with different pore diameters and the results showed that the maximum number of particles entrapment was achieved with the 2 μm pore and 10 mM ionic strength solution (Fig. 6a).

However, we have noticed an inconsistency in the entrapment yield using pipettes with the same pore size but different geometries, similar to the report made by Klenerman and co-workers^38^. Although, it is difficult to completely eliminate the variation in pipette’s geometry utilizing the current fabrication technique, the inconsistency can be justified by investigating the key geometrical factors such as the tapered angle of the pipettes’ wall which have a crucial role on particles entrapment. Microscopic examination of the pipettes showed the tapered angle variations from 6.0° to 10.2° for 500 nm pipettes (Table S1), 10.6° to 13.5° for 1 μm pipettes (Table S2), and 14.6° to 21.6° for 2 μm pipettes (Table S3). To further investigate the effect of the tapered angle, another set of experiments were run using a 1 μm pipette with a larger tapered angle (15.1°). The results indicated a significant increase in particles entrapment as the angle increased from 11.1° to 15.1° (Fig. S5). This could be justificed by the reduction of the EOF at the center of the pipette and thus, the reduction of the repeling force applied to the particles as the tapered angle increased. Although, our observations showed an increase in the entrapment yeild with respect to the increased of the tapered angel, more systematic analysis is required in future to comprehensively investigate the effect of the pipettes’ geometry on particles entrapment.

### The effect of run time on particles entrapment

To investigate the maximum entrapment yield of the system, the experiments were conducted in longer time intervals using a 2 μm pipette and 10 mM KCl solution. The semi-quantitative analysis of fluorescence intensity of the trapped particles was conducted to estimate the number of trapped particles after one hour of 10 V/cm positive bias applied at the base of the pipette (Figs 6b and S6). The number of trapped particles was calculated as 386 after 100 seconds, 1390 after 5 minutes, 2400 after 10 minutes, 4500 after 30 minutes, and 5300 after 1 hour of applied potential. There was approximately 3.6 fold increase in the number of particles at the tip from 100 seconds to 5 minutes, ~1.8 fold increase after 30 minutes, and from 30 minutes to 1 hour there was around 1.1 fold increase in the number of particles. The trapping rate was fast within the first 5 minutes of applied potential, approximately 4.6 particles/sec. However, the rate decreased significantly to 2.5 particles/sec after 30 minutes and 1.5 particles/sec after 1 hour (Fig. 6b). The decrease in the entrapment rate and the concentration of the particles is because of the net EK force drop at the tip as the beads accumulated and thus, saturated the trapping zone. Also, the particles accumulation by the tip diminished the fluid flow through the pipette, which slowed down the motion of the particles from bulk toward the trapping zone. Although, the entrapment yield of a single pore is quite low (~0.8%), the improvement can be achieved in future by fabricating an array of pores on a glass substrate to multiplex the entrapment events.

### Selective entrapment of liposomes suspended in the PBS solution

**Figure 7:**
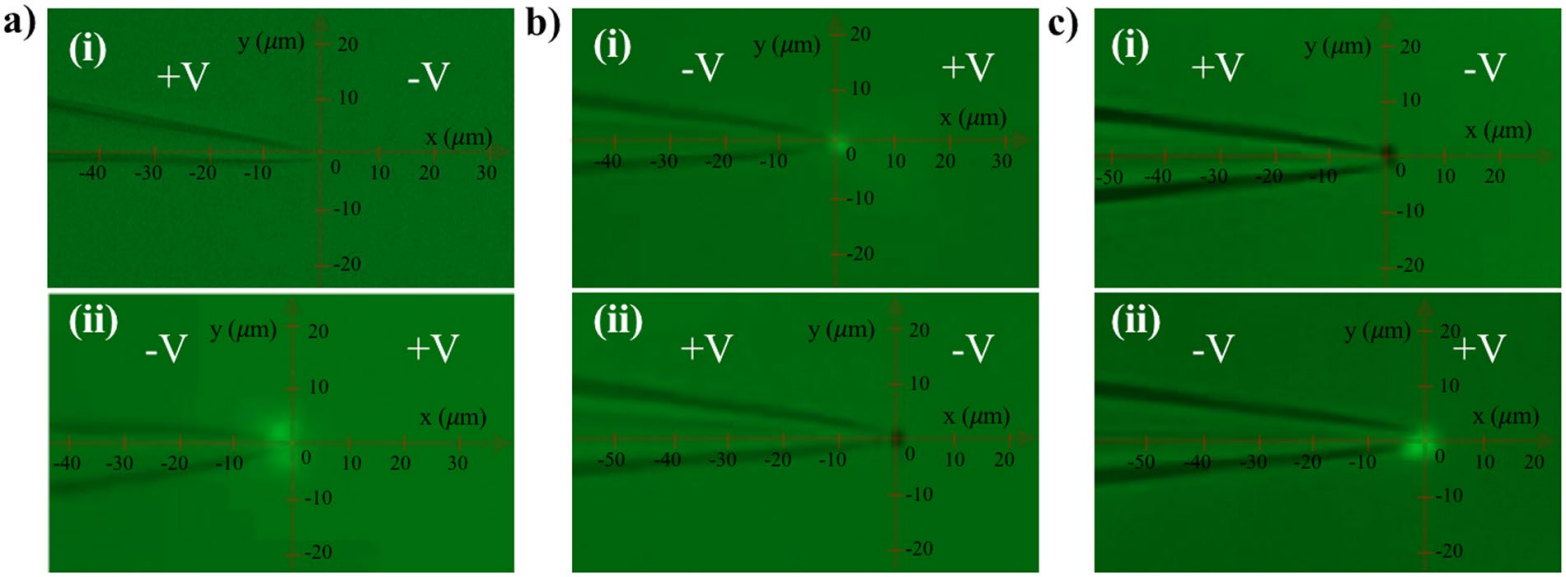
(**a**) Entrapment of 100 nm fluorescently-tagged liposomes suspeneded in the PBS solution using a 1 μm pipette and 10 V/cm applied E-field. (i) The results after 100 seconds of applied positive bias at the base. (ii) The results after 100 seconds of applied negative bias at the base. (**b**) Selective entrapment of 100 nm flourescently-tagged liposomes and 510 nm non-fluorescent COOH-PS beads suspended in the PBS solution. (i) The results after 100 seconds of applied negative bias at the base of the pipette. (ii) The results after 100 seconds of the voltage polarity reversal to positive bias at the base. (**c**) Selective entrapment of 100 nm fluorescently-tagged liposomes and 510 nm non-fluorescent COOH-PS beads suspended in PBS solution. (i) The results after 100 seconds of applied positive bias at the base of the pipette. (ii) The results after 100 seconds of the voltage polarity reversal to negative bias at the base.

Liposomes are nanoscale sacs made up of lipid bilayers that have been widely studied as a model of biological membrane vesicles over the past few decades^45–47^. To investigate the capability of our device to trap extracellular vesicles, 100 nm fluorescently labeled artificial liposomes re-suspended in PBS solution were utilized as a model system. Figure 7a shows the trapping results of liposomes using a 1 μm pipette after 100 seconds of applied voltage (10 V/cm). Panel (i) illustrates that as the positive bias was applied at the base of the pipette, no liposome was trapped. However, as the voltage polarity was reversed to the negative bias, liposomes were trapped by the tip (Fig. 7a(ii)). Since liposomes have lower zeta potential (~ −8 mV) and relatively smaller size (100 nm) compared to the COOH-PS beads, the EP force acting on the liposomes is much smaller than the EP force applied to the beads. Also, the magnitude of the DEP force induced on the liposomes becomes smaller due to the reduction of their size (Eq. (3)) and therefore, the electroosmosis is the dominant force, which attracts the vesicles toward the pore. Moreover, the conductivity of the liposome’s membrane made of DOPC and cholesterol has been reported to be less than 1 nS/m^48^. Thus, the conductivity of the liposomes used in this study was calculated to be less than 1 × 10^−2^ μS/cm, based on the dielectric multi-shell model^48^, and as a result, liposomes would be under negative DEP force when suspended in the PBS solution. We can conclude that, as the negative bias was applied at the base, EOF attracted the liposomes toward the trapping zone while the negative DEP and EP pushed them away from the tip, preventing them from translocating through the pipette which resulted in their entrapment by the tip.

To further investigate the effect of voltage polarity on vesicles entrapment, selective entrapment of liposomes was achieved in PBS solution containing COOH-PS beads. The results show that as the negative bias was applied at the base, liposomes were selectively trapped (Fig. 7b(i) and Video S2) and as the voltage polarity was reversed, the non-fluorescent COOH-PS beads were trapped while the liposomes were released (Fig. 7b(ii) and Video S2). The same results were obtained as the positive bias was initially applied followed by the reversal of the voltage polarity (Fig. 7c).

### Entrapment of exosomes suspended in the PBS solution

**Figure 8:**
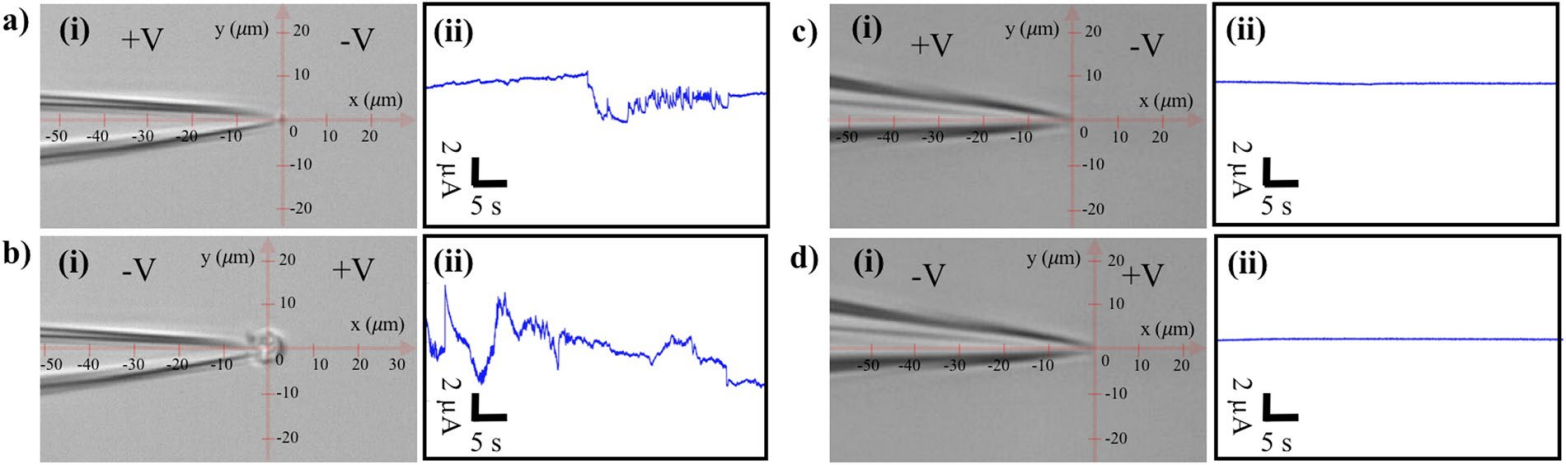
(**a**) (i) Trapping results of plasma-driven exosomes re-suspended in the PBS solution with positive potential applied at the base of the pipette after 100 seconds. (**a**) (ii) Corresponding conductance changes across the pore. (**b**) (i) Trapping results of exosomes after reversal of the voltage polarity to the negative potential at the base of the pipette after 100 seconds. (**b**) (ii) Corresponding conductance changes across the pore. (**c**–**d**) The blank control experiments without exosomes and the corresponding conductance measurements across the pores. The diameter of the pipette was 1 μm and the applied E-field was 10 V/cm.

Exosomes are small membrane vesicles, 30–150 nm in size, released by cells into the extracellular space via the exocytosis pathway^49,50^. Since exosomes contain important gene regulatory content such as proteins, microRNAs (miRs), and messenger RNAs (mRNAs)^51–54^, it is believed that they can play an important role as biomarkers for diagnosis and new semi-synthetic drug delivery vehicles for personalized therapy^55–57^. However, one of the major challenges in the field of extracellular vesicles is the difficulty in their selective entrapment from body fluids or cell culture media due to their small size. Here, we investigate the capability of our system to trap plasma-driven exosomes re-suspended in the PBS solution using our label-free nanopipette DEP device. Figure 8a and b show the results of exosome entrapment experiments after 100 seconds of 10 V/cm applied bias across a 1 μm pipette. Figure 8a(i) illustrates that only a few exosomes were trapped with the applied positive bias at the base; and more significant entrapment was observed after the voltage polarity was reversed (Fig. 8b(i) and Video S3). Since exosomes have a similar zeta potential (~ −11 mV) and size as liposomes, they were trapped more significantly as the negative voltage polarity was applied at the base of the pipette. The conductance changes were observed under both voltage polarities as a result of partial blockade of the pore by the trapped exosomes at the tip (Fig. 8a(ii),b(ii)). However, the conductance variations represent a qualitative indication of the entrapment events and there is no significant correlation between the shape of the electrical signal and the number of trapped exosomes. A control experiment with blank PBS solution, containing no exosome, was performed to ensure no false positive entrapment results (Fig. 8c,d). The exosome entrapment was repeated as the sequence of voltage polarity was reversed: initially negative bias was applied at the base, followed by the positive bias. The results showed the same entrapment trends as observed in Fig. 8a (data not shown).

## Conclusions

A new insulator-based nanopipette-DEP device with low applied DC field for rapid entrapment of nanoparticles and nano-vesicles was presented. The simulation and experimental results indicated that the entrapment was governed by the net electrokinetic force induced on the particles; and the entrapment mechanism and yield are dependent on the polarity of the applied voltage, the size and the geometry of pipette, and the ionic strength of the solution. The best trapping condition was explored under 10 mM KCl solution using a 2 μm pipette and 10 V/cm positive bias applied at the base of the pipette. The entrapment yield enhanced with time and reached a plateau after approximately 40 minutes. However, the rate of entrapment decreased after 10 minutes of applied potential due to the screening effect of the trapped particles, on the EK forces at the trapping zone. To illustrate the biomedical application of the device, exosomes extracted from plasma of the healthy donors and liposomes re-suspended in the PBS solution were successfully trapped under the applied negative potential. The results illustrate the sensitivity of this device to the nanoparticles’ size and conductivity. In addition, selective entrapment of liposomes and COOH-PS beads suspended in the PBS solution were obtained by switching the voltage polarity across the pipette. In future, this scheme can be further optimized as a label-free tool with spatiotemporal resolution to facilitate the selective entrapment of extracellular vesicles from cell culture medium or biofluids by applying a combination of DC and AC fields.

## Methods

### Materials

All chemicals were purchased from Sigma-Aldrich (St. Louis, MO, USA) unless otherwise noted. Silicone elastomer base and curing agents were purchased from Dow Corning (Elizabethtown, KY, USA). Fluorescently tagged and non-fluorescently tagged COOH-PS beads were obtained from Bangs Laboratories, Inc. (Fisher, IN, USA). Lyophilized exosomes from plasma of healthy donors were obtained from HansaBioMed Life Sciences Ltd. (Tallinn, Estonia). 100 nm NBD-DHPE labeled fluorescent liposomes were purchased from FormuMax Scientific Inc. (Sunnyvale, CA, USA). The liposome membrane was composed of phospholipid DOPC, cholesterol and NBD-DHPE fluorescent dye with a molar ratio 54:45:1. Borosilicate filaments were purchased from Sutter Instrument (Novato, CA, USA).

### Preparation and characterization of particles

Potassium chloride (KCl) solutions at pH of 7.0 with different concentrations (500 mM, 100 mM, 10 mM, and 1 mM) were prepared and the PBS solution was diluted 10 times at pH 7.4. The conductivity of the solutions was measured as: 16.22 mS/cm for 1xPBS solution, 58.8 mS/cm for 500 mM KCl, 13.94 mS/cm for 100 mM KCl, 3.00 mS/cm for 10 mM KCl solution, and 1.10 mS/cm for 1 mM KCl solution using a conductivity meter (Oakton Cond 6+).

COOH-PS beads with 510 nm diameters were re-suspended into different electrolyte solutions to the final concentration of 1.37 × 10^7^/mL. The conductivity of COOH-PS beads was calculated based on the report by Morgan *et al*.^58^, as 15.6 μS/cm. The zeta potential of the particles dispersed in different electrolyte solutions at 25 °C were measured at least 3 times using the Zetasizer-NanoBrook Omni (Brookhaven Instruments) (Table S4). Fluorescently labeled liposomes with 100 nm diameters were re-suspended into 1x PBS solution at a final concentration of 1x 10^11^/mL. Lyophilized exosomes were reconstructed in 1x PBS solution at the final concentration of 1 × 10^8^/mL.

### Device fabrication and setup

Nanopipettes with different diameters (500 nm, 1000 nm and 2000 nm) were fabricated with the laser assisted puller, Sutter-2000 using the appropriate program (Table S5). The diameters of pipettes were approximated by comparing their conductance with the conductance of the same sized pipettes purchased from World Precision Instruments, Inc.

The device fabrication and the data acquisition were obtained as previously reported by our group^59^. To ensure the pore’s diameter consistency, the ionic current across each nanopipette was measured in each experiment and reported in Tables S1–S3. 50 μL of electrolyte solutions containing nanoparticles were added in the chamber facing the pipette’s tip and an electric potential was applied while the ionic current was measured. The motion of the particles was simultaneously monitored and recorded using an inverted fluorescent microscope, the Nikon Eclipse TE2000-S, equipped with a high-resolution camera, Andor NeoZyla 5.5, at a capturing frequency of 100 frames per second. Each experiment was repeated at least 3 times.

### Finite Element Analysis of the flow behavior

Using the finite-element software, COMSOL Multiphysics 5.2a, the distribution of the EP, DEP, and EOF drag force acting on the PS-COOH beads was determined as the applied voltage, the salt concentration and the pipettes’ pore size were systematically varied. The system being modeled consisted of a 1-D model where Poisson’s and Nernst-Planck Equations were solved for potential variation and concentration distribution of ionic species along the boundary of the system and further served as the Dirichlet boundary conditions for the 2-D model, emulating the pipette setup. The 2-D axisymmetric design, comprised of the borosilicate pipette suspended in a circular reservoir filled with monovalent buffered salt, was constructed. Boundary conditions corresponding to the solution obtained from the Poisson-Boltzmann equation for electric potential were applied. The conditions established that the electric potential did not diverge anywhere and the gradient of this potential on the pipette surface varied with the change in surface charge density^44^.

The model computed a combination of multiple physical phenomena pertaining to different aspects of the system: ‘Electrostatics (es)’ catered to the surface charge and voltage related analysis, ‘Creeping flow (spf)’ was solved for the study of incompressible and non-isothermal flow along the glass walls of the pipette, and ‘Transport of Diluted Species (tds)’ was incorporated for the migration of ionic species with the applied fields. The solution of the system provided the electric field and gradient of the square of electric field along the entire pipette length (Fig. S7). Further, a comprehensive qualitative analysis of different force distributions along the entire pipette’s length and the region outside was conducted as line graphs similar to a study by Clarke *et al*.^39^. Each force was mathematically represented by an equation in the process and was plotted along the pipette’s length in the axisymmetric model.

Different physical conditions in the experimental setup gave rise to a set of phenomena and eventually introduced the forces in the system. The negatively charged microspheres interacted with the applied electric field and experience the EP force under the applied bias. This force can be mathematically computed using equation (1):

1 $${F}_{EP}=6\pi \eta r{\mu }_{EP}E$$

where $\eta$ is the viscosity of the solution, E is the applied electric field strength, ${\mu }_{EP}$ is electrophoretic mobility, which was measured empirically using the Zetasizer.

The electroosmotic mobility of the particles was evaluated using equation (2):

2 $${\mu }_{EOF}=-\,({\varepsilon }_{m}\,\zeta )/4\pi \eta $$

where ${\varepsilon }_{m}$ is the medium permittivity, and $\zeta$ is the zeta-potential (ZP) of the glass walls of the micropipette. The zeta-potential for the glass pipette can be estimated using Graham’s equation^60^, which relates the ZP to the estimated surface charge density of the micropipette. The surface charge density of the borosilicate pipette was evaluated to be −0.02 C/m^2^ by Behrens and Grier^61^.

The zeta values of the pipette were estimated to be −123, −66.8, −26.3, and −12.2 mV for 1, 10, 100, and 500 mM buffered salt concentrations at pH 7.0, respectively. Also, the ZP of the glass wall was assumed to be constant over the entire surface of the pipette in the model.

The DEP force on particles at the near tip region was computed using equation (3):

3 $$\,{F}_{DEP}=2\pi {r}^{3}{\varepsilon }_{m}Re({f}_{CM}){\nabla }{E}^{2}$$

where *r* is the particle radius, and $Re({f}_{CM})$ is the real part of Clausius-Mossotti factor represented below:

4 $$Re({f}_{CM})=({\varepsilon }_{p}^{\ast }-{\varepsilon }_{m}^{\ast })/({\varepsilon }_{p}^{\ast }+2{\varepsilon }_{m}^{\ast })$$

where ${\varepsilon }_{p}^{\ast }$ and $\,{\varepsilon }_{m}^{\ast }\,\,$are the complex permittivity of the particle and the medium. The complex permittivity is expressed by ${\varepsilon }^{\ast }=\varepsilon -(j\sigma /\omega )$, where $\varepsilon$ is the real permittivity, $\sigma$ is the conductivity, and $\omega$ is the angular frequency of the applied electric field^62^.

The Clausius-Mossotti factor under DC field can be represented as equation (5):^34^

5 $$Re({f}_{CM})=({\sigma }_{p}-{\sigma }_{m})/({\sigma }_{p}+2{\sigma }_{m})$$

where ${\sigma }_{m}$ and ${\sigma }_{p}$ are the medium and particle conductivities. By combining these three forces, a cumulative net force was plotted to evaluate the overall trend of the forces in place by mathematically adding the vector components from the equations along the entire length of the pipette^43^.

We have neglected the effect of electro-thermal flow (ETF) in our system since the ETF is mostly significant under the high-frequency AC voltage conditions^63,64^. In previous reports, the maximum electro-thermal flow velocity at the tip region was calculated to be in the order of 10^−4^ m/s^65,66^. However, based on our simulation results, the fluid flow velocity in our system is around 1 × 10^−1^ m/s which is three orders of magnitude higher than the reported ETF velocity due to the presence of EOF in our system. In addition, the electro-thermal force can be calculated as^67^:

6 $${F}_{T}=({k}_{B}\,{T}_{R})/(2r)$$

where ${k}_{B}$ is the Boltzmann constant, ${T}_{R}$ is the room temperature and *r* is the particle radius. In our system, the electro-thermal force is calculated as approximately 10^−4^ nN which is three orders of magnitude lower than the other three dominant EK forces.

### Data analysis

The fluorescence intensity of the trapped particles was semi-quantified using the NIS-Elements Advanced Research software (Nikon Instruments). The number of trapped particles was estimated by dividing the total fluorescent intensity of the trapped particles by the intensity of an individual particle.

An open source particle tracking software, Tracker 4.9.8, was used to measure the tapered angle of the pipettes and track the trajectories of the particles. Real time recorded footage during each experiment was run frame by frame. A frame of reference axes was established with its origin at the tip and a calibration length was marked to provide a reference length scale for the moving particles. At each frame, a chosen particle of interest was manually marked for its coordinate locations until it was trapped at the tip. Three beads were tracked for each experimental condition, and the process was repeated at least three times for each bead. Once the coordinate data corresponding to the motion of beads was obtained, instantaneous velocities were computed at each frame.

## Electronic supplementary material

Supplimentary Information

Vidoe S1

Video S2

Video S3

## References

[1] Ashkin A, Dziedzic J, Bjorkholm J, Chu S (1986). Observation of a single-beam gradient force optical trap for dielectric particles. Optics letters.

[2] Shao B, Zlatanovic S, Ozkan M, Birkbeck AL, Esener SC (2006). Manipulation of microspheres and biological cells with multiple agile VCSEL traps. Sensors and Actuators B: Chemical.

[3] Monat C, Domachuk P, Eggleton B (2007). Integrated optofluidics: A new river of light. Nature photonics.

[4] McCloskey KE, Chalmers JJ, Zborowski M (2003). Magnetic cell separation: characterization of magnetophoretic mobility. Analytical chemistry.

[5] Liu C, Lagae L, Borghs G (2007). Manipulation of magnetic particles on chip by magnetophoretic actuation and dielectrophoretic levitation. Applied physics letters.

[6] Jung J, Han K-H (2008). Lateral-driven continuous magnetophoretic separation of blood cells. Applied Physics Letters.

[7] Nilsson A, Petersson F, Jönsson H, Laurell T (2004). Acoustic control of suspended particles in micro fluidic chips. Lab on a Chip.

[8] Shi J, Huang H, Stratton Z, Huang Y, Huang TJ (2009). Continuous particle separation in a microfluidic channel via standing surface acoustic waves (SSAW). Lab on a Chip.

[9] Guldiken R, Jo MC, Gallant ND, Demirci U, Zhe J (2012). Sheathless size-based acoustic particle separation. Sensors.

[10] Washizu M, Suzuki S, Kurosawa O, Nishizaka T, Shinohara T (1994). Molecular dielectrophoresis of biopolymers. IEEE Transactions on Industry Applications.

[11] Lapizco‐Encinas BH, Simmons BA, Cummings EB, Fintschenko Y (2004). Insulator‐based dielectrophoresis for the selective concentration and separation of live bacteria in water. Electrophoresis.

[12] Morgan H, Hughes MP, Green NG (1999). Separation of submicron bioparticles by dielectrophoresis. Biophysical journal.

[13] Asbury CL, Diercks AH, van den Engh G (2002). Trapping of DNA by dielectrophoresis. Electrophoresis.

[14] Asbury CL, Engh VD (1998). G. Trapping of DNA in nonuniform oscillating electric fields. Biophysical Journal.

[15] Schnelle T, Müller T, Hagedorn R, Voigt A, Fuhr G (1999). Single micro electrode dielectrophoretic tweezers for manipulation of suspended cells and particles. Biochimica et Biophysica Acta (BBA)-General Subjects.

[16] Huang Y (2001). Electric manipulation of bioparticles and macromolecules on microfabricated electrodes. Analytical chemistry.

[17] Modarres, P. & Tabrizian, M. Alternating current dielectrophoresis of biomacromolecules: The interplay of electrokinetic effects. *Sensors and Actuators B: Chemical* (2017).

[18] Pethig R (2010). Dielectrophoresis: Status of the theory, technology, and applications. Biomicrofluidics.

[19] Voldman J (2006). Electrical forces for microscale cell manipulation. Annu. Rev. Biomed. Eng..

[20] Jubery TZ, Srivastava SK, Dutta P (2014). Dielectrophoretic separation of bioparticles in microdevices: A review. Electrophoresis.

[21] Lapizco‐Encinas BH, Rito‐Palomares M (2007). Dielectrophoresis for the manipulation of nanobioparticles. Electrophoresis.

[22] Alazzam A, Stiharu I, Bhat R, Meguerditchian AN (2011). Interdigitated comb‐like electrodes for continuous separation of malignant cells from blood using dielectrophoresis. Electrophoresis.

[23] Li H, Bashir R (2002). Dielectrophoretic separation and manipulation of live and heat-treated cells of Listeria on microfabricated devices with interdigitated electrodes. Sensors and Actuators B: Chemical.

[24] Wang L (2009). Dual frequency dielectrophoresis with interdigitated sidewall electrodes for microfluidic flow‐through separation of beads and cells. Electrophoresis.

[25] Germishuizen WA (2003). Selective dielectrophoretic manipulation of surface-immobilized DNA molecules. Nanotechnology.

[26] Hölzel R, Calander N, Chiragwandi Z, Willander M, Bier FF (2005). Trapping single molecules by dielectrophoresis. Physical review letters.

[27] Bakewell, D. J., Hughes, M. P., Milner, J. J. & Morgan, H. In *Engineering in Medicine and Biology Society*, *1998*. *Proceedings of the 20th Annual International Conference of the IEEE*. 1079–1082 (1998).

[28] Zheng L, Brody JP, Burke PJ (2004). Electronic manipulation of DNA, proteins, and nanoparticles for potential circuit assembly. Biosensors and Bioelectronics.

[29] Yasukawa T, Suzuki M, Shiku H, Matsue T (2009). Control of the microparticle position in the channel based on dielectrophoresis. Sensors and Actuators B: Chemical.

[30] Green NG, Morgan H (1997). Dielectrophoretic separation of nano-particles. Journal of Physics D: Applied Physics.

[31] Luo J, Abdallah BG, Wolken GG, Arriaga EA, Ros A (2014). Insulator-based dielectrophoresis of mitochondria a. Biomicrofluidics.

[32] Kang Y, Li D, Kalams SA, Eid JE (2008). DC-Dielectrophoretic separation of biological cells by size. Biomedical microdevices.

[33] Pethig R (2010). Review article—dielectrophoresis: status of the theory, technology, and applications. Biomicrofluidics.

[34] Ozuna‐Chacón S, Lapizco‐Encinas BH, Rito‐Palomares M, Martínez‐Chapa SO, Reyes‐Betanzo C (2008). Performance characterization of an insulator‐based dielectrophoretic microdevice. Electrophoresis.

[35] Chen D, Du H (2010). A microfluidic device for rapid concentration of particles in continuous flow by DC dielectrophoresis. Microfluidics and Nanofluidics.

[36] Ying L, Bruckbauer A, Rothery AM, Korchev YE, Klenerman D (2002). Programmable delivery of DNA through a nanopipet. Analytical chemistry.

[37] Ying L (2004). Frequency and voltage dependence of the dielectrophoretic trapping of short lengths of DNA and dCTP in a nanopipette. Biophysical journal.

[38] Clarke RW, White SS, Zhou D, Ying L, Klenerman D (2005). Trapping of proteins under physiological conditions in a nanopipette. Angewandte Chemie International Edition.

[39] Clarke RW, Piper JD, Ying L, Klenerman D (2007). Surface conductivity of biological macromolecules measured by nanopipette dielectrophoresis. Physical review letters.

[40] Song Y, Sonnenberg A, Heaney Y, Heller MJ (2015). Device for dielectrophoretic separation and collection of nanoparticles and DNA under high conductance conditions. Electrophoresis.

[41] Freedman, K. J. *et al*. Nanopore sensing at ultra-low concentrations using single-molecule dielectrophoretic trapping. *Nature communications* **7** (2016). 10.1038/ncomms10217 PMC4729827 26732171

[42] Hunt T, Westervelt R (2006). Dielectrophoresis tweezers for single cell manipulation. Biomedical microdevices.

[43] Rempfer G (2016). Selective Trapping of DNA Using Glass Microcapillaries. Langmuir.

[44] Laohakunakorn N, Thacker VV, Muthukumar M, Keyser UF (2014). Electroosmotic flow reversal outside glass nanopores. Nano letters.

[45] Goyal G, Darvish A, Kim MJ (2015). Use of solid-state nanopores for sensing co-translocational deformation of nano-liposomes. Analyst.

[46] Bangham, A., Hill, M. & Miller, N. In *Methods in membrane biology* 1–68 (Springer, 1974).

[47] Martin F, MacDonald R (1974). Liposomes can mimic virus membranes. Nature.

[48] Chan KL, Gascoyne PR, Becker FF, Pethig R (1997). Electrorotation of liposomes: verification of dielectric multi-shell model for cells. Biochimica et Biophysica Acta (BBA)-Lipids and Lipid Metabolism.

[49] Kalra H, Drummen GP, Mathivanan S (2016). Focus on extracellular vesicles: introducing the next small big thing. International journal of molecular sciences.

[50] Keller S, Sanderson MP, Stoeck A, Altevogt P (2006). Exosomes: from biogenesis and secretion to biological function. Immunology letters.

[51] Johnstone RM (2006). Exosomes biological significance: a concise review. Blood Cells, Molecules, and Diseases.

[52] Valadi H (2007). Exosome-mediated transfer of mRNAs and microRNAs is a novel mechanism of genetic exchange between cells. Nature cell biology.

[53] Zhang J (2015). Exosome and exosomal microRNA: trafficking, sorting, and function. Genomics, proteomics & bioinformatics.

[54] van Hoof A, Parker R (1999). The exosome: a proteasome for RNA?. Cell.

[55] Munson P, Shukla A (2015). Exosomes: potential in cancer diagnosis and therapy. Medicines.

[56] Conlan RS, Pisano S, Oliveira MI, Ferrari M, Pinto IM (2017). Exosomes as Reconfigurable Therapeutic Systems. Trends in molecular medicine.

[57] Wang J, Zheng Y, Zhao M (2017). Exosome-based cancer therapy: implication for targeting cancer stem cells. Frontiers in pharmacology.

[58] Cui L, Holmes D, Morgan H (2001). The dielectrophoretic levitation and separation of latex beads in microchips. Electrophoresis.

[59] Zhang Y, Rana A, Stratton Y, Czyzyk-Krzeska MF, Esfandiari L (2017). Sequence-Specific Detection of MicroRNAs Related to Clear Cell Renal Cell Carcinoma at fM Concentration by an Electroosmotically Driven Nanopore-Based Device. Analytical chemistry.

[60] Arjmandi N, Van Roy W, Lagae L, Borghs G (2012). Measuring the electric charge and zeta potential of nanometer-sized objects using pyramidal-shaped nanopores. Analytical chemistry.

[61] Behrens SH, Grier DG (2001). The charge of glass and silica surfaces. The Journal of Chemical Physics.

[62] Dielectrophoresis, H. P. (Cambridge university press Cambridge, 1978).

[63] Sasaki N, Kitamori T, Kim HB (2012). Fluid mixing using AC electrothermal flow on meandering electrodes in a microchannel. Electrophoresis.

[64] Ramos A, Morgan H, Green NG, Castellanos A (1998). Ac electrokinetics: a review of forces in microelectrode structures. Journal of Physics D: Applied Physics.

[65] Khoshmanesh K (2011). Dynamic analysis of drug-induced cytotoxicity using chip-based dielectrophoretic cell immobilization technology. Analytical chemistry.

[66] Tang S-Y (2014). High resolution scanning electron microscopy of cells using dielectrophoresis. PloS one.

[67] Barik A (2017). Graphene-edge dielectrophoretic tweezers for trapping of biomolecules. Nature communications.

