# Supplementary material for: A low voltage nanopipette dielectrophoretic device for rapid entrapment of nanoparticles and exosomes extracted from plasma of healthy donors
Source: Sci Rep. 2018 Apr 30;8:6751. doi: 10.1038/s41598-018-25026-2 (PMC5928082; doi:10.1038/s41598-018-25026-2)
Supplement: Supplementary file 1 — Supplimentary Information [file 41598_2018_25026_MOESM1_ESM.pdf]

Supplementary Information

A low voltage nanopipette dielectrophoretic device for rapid entrapment of nanoparticles and exosomes extracted from plasma of healthy donors

Leilei Shi[1], Ankit Rana[1], Leyla Esfandiari*[1,2]

Department of Electrical Engineering and Computer Science[1]

Department of Biomedical Engineering[2]

College of Engineering and Applied Science, University of Cincinnati, Ohio 45221, United States, E-mail: esfandla@ucmail.uc.edu, Fax: 513-556-732

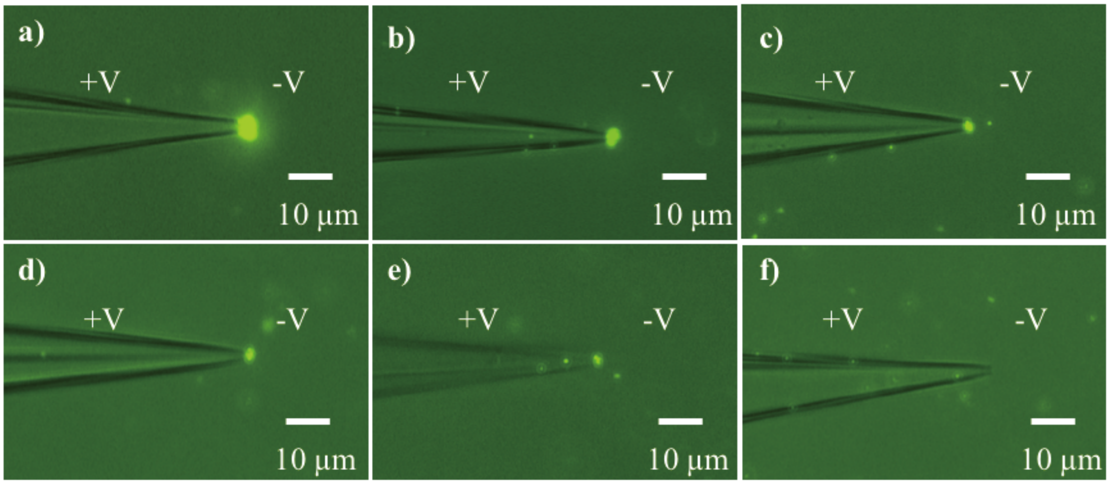

Figure S1. The entrapment results of 510 nm COOH-PS nanoparticles suspended in 10 mM KCl pH 7.0 using a 1 μm pipette under different magnitude of applied potential after 100 seconds. From a) to f), the applied electric field was 10, 5, 3.33, 1.67, 1, and 0.67V/cm.

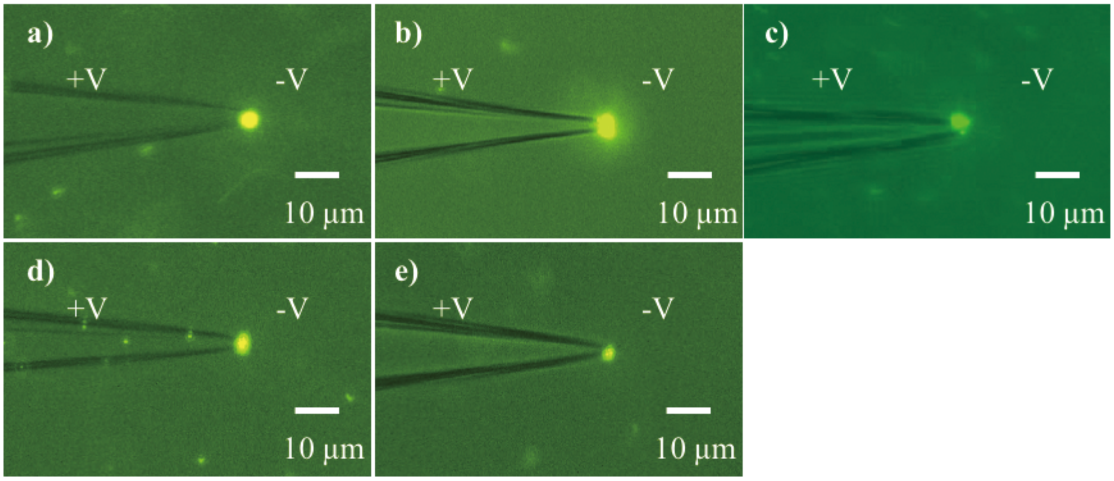

Figure S2. The entrapment results of 510 nm fluorescent COOH-PS beads suspended in different salt solutions using a 1 μm pipette under 10 V/cm after 100 seconds. From a) to e), the salt concentration was 1, 10, 100, 500 mM KCl, and PBS.

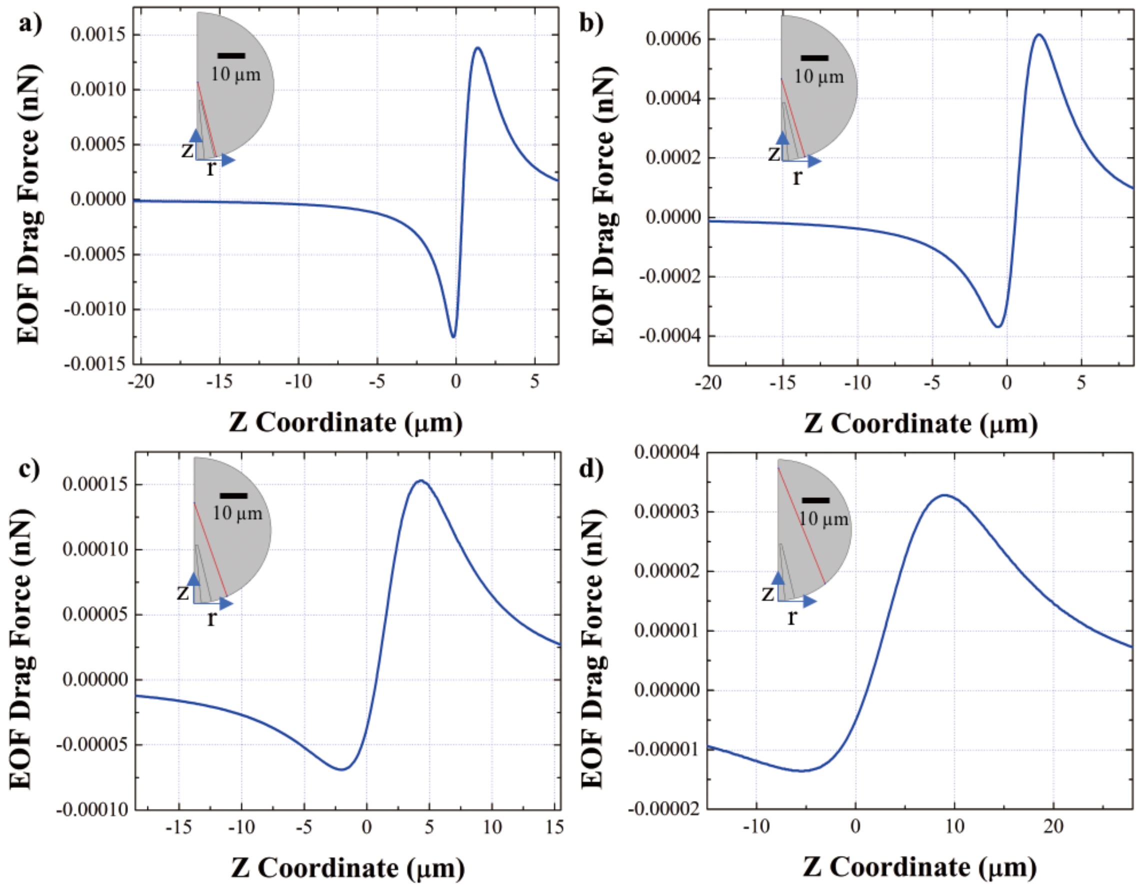

Figure S3. The trend in EOF magnitude acting on the 510 nm COOH-PS particles suspended in 10 mM KCl with respect to the disctance of the particles from the pipettes' wall. The inset figure is the 2D axisymmetric model of the nanopipette and the EOF along the red tracer is plotted. A 10 V/cm E-field was applied at the base of the 1 μm pipette.

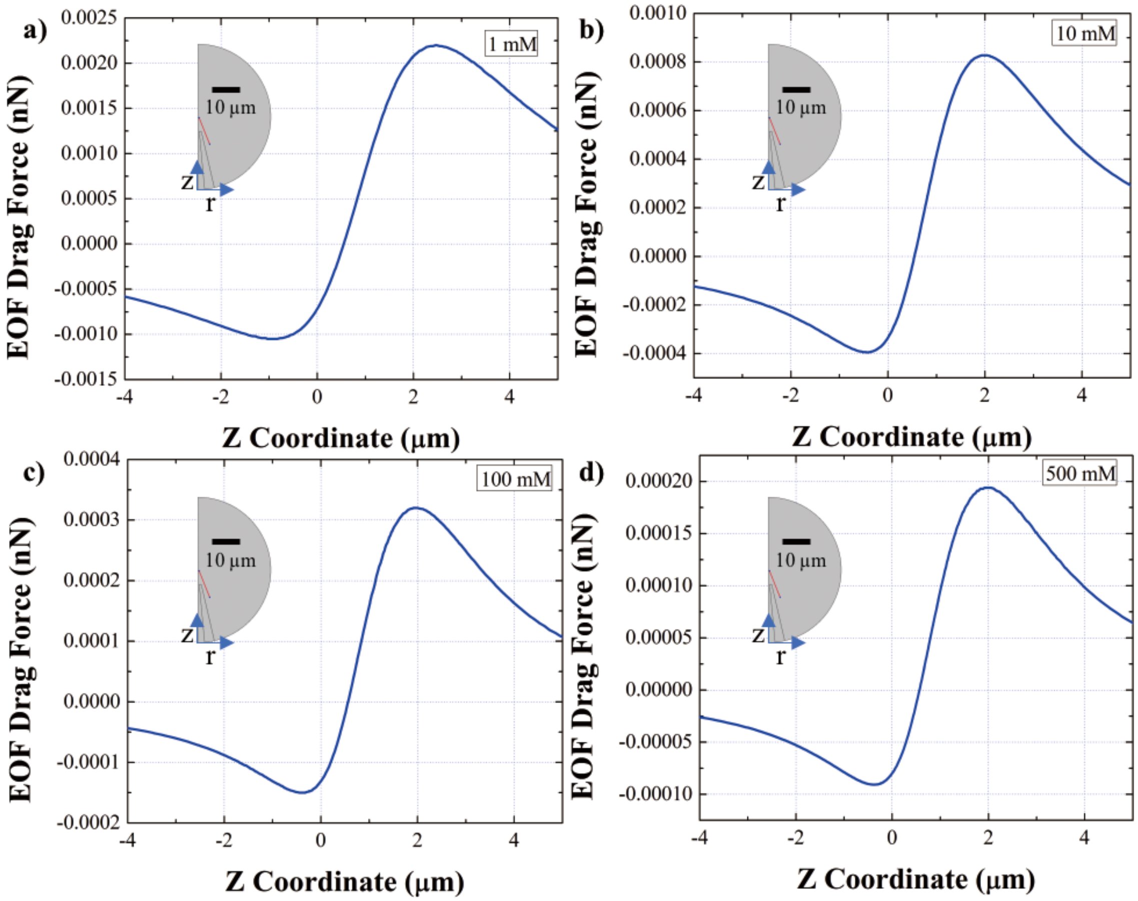

Figure S4. The EOF distribution along the contour as the ionic strength varies as: a) 1 mM KCl, b) 10 mM KCl, c) 100 mM KCl, d) 500 mM KCl. The inset figure is the 2D axisymmetric model of the nanopipette and the EOF where the red tracer is plotted. The particles were 510 nm COOH-PS beads, and a 10V/cm E-field was applied at the base of the 1 µm pipette.

Table S1. Summary of 500 nm pipettes' tapered angles and the open pore conductance under 10 V/cm in different ionic strength solutions.

Solution	Angle (°)	R_{open} (Ω)
1 mM KCl	7.0	8.11E+08
	9.1	6.25E+08
	7.4	5.77E+08
10 mM KCl	8.0	3.61E+08
	6.6	5.00E+08
	6.0	3.00E+08
100 mM KCl	10.2	3.33E+07
	8.0	4.69E+07
	7.7	5.36E+07

500 mM KCl	8.1	6.85E+06
	7.5	1.07E+07
	8.8	7.56E+06
PBS	7.0	2.10E+07
	10.1	1.27E+07
	7.6	1.89E+07

Table S2. Summary of 1 μm pipettes' tapered angle and open pore conductance under different electric field and different ionic strength solutions.

Solution	E-field (V/cm)	Angle (°)	R_{open} (Ω)
1 mM KCl	10	12.5	3.49E+07
		12.3	4.23E+07
		11.2	7.89E+07
10 mM KCl	10	13.5	3.03E+07
		12.7	3.13E+07
		12.5	2.78E+07
100 mM KCl	10	12.9	3.00E+06
		13.2	2.44E+06
		11.1	2.00E+06
500 mM KCl	10	11.4	8.82E+05
		13.5	6.82E+05
		13.5	7.61E+05
PBS	10	10.6	2.13E+06
		12.2	2.31E+06
		12.0	4.11E+06
10 mM KCl	5	11.7	2.42E+07
		13.0	2.68E+07
		12.2	2.59E+07
10 mM KCl	3.33	12.3	2.86E+07
		12.3	2.22E+07
		13.2	2.08E+07

10 mM KCl	1.67	12.1	2.63E+07
		12.7	2.27E+07
		11.3	2.27E+07
10 mM KCl	1	11.9	2.50E+07
		12.4	3.00E+07
		13.1	1.43E+07

Table S3. Summary of 2 μm pipettes' tapered angle and open pore conductance under 10 V/cm in different ionic strength solutions.

Solution	Angle (°)	R_{open} (Ω)
1 mM KCl	17.9	2.27E+07
	21.6	1.60E+07
	16.5	1.86E+07
10 mM KCl	16.3	8.36E+06
	15.0	9.38E+06
	17.6	8.29E+06
100 mM KCl	14.6	1.26E+06
	18.5	1.05E+06
	19.9	1.19E+06
500 mM KCl	18.5	2.36E+05
	17.3	2.04E+05
	19.6	2.00E+05
PBS	17.2	1.05E+06
	17.1	7.01E+05
	15.2	8.57E+05

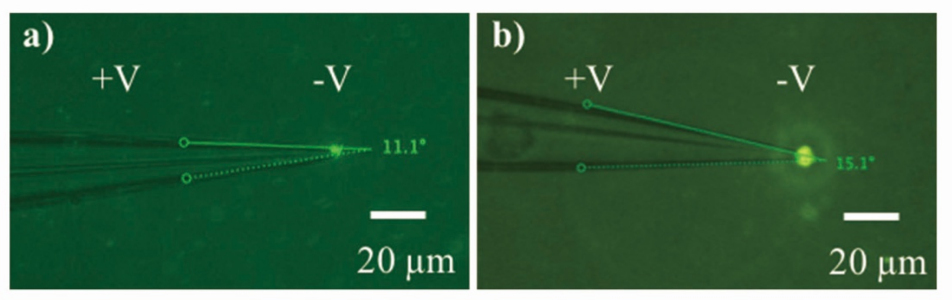

Figure S5. The trapping results of 510 nm COOH-PS beads in 10 mM KCl at pH 7.0 with 1μm pipette's that have different tapered angles under 10 V/cm. a) The trapping result with a tapered angle of 11.1° after 100 seconds. b) The trapping result with a tapered angle of 15.1° after 100 seconds.

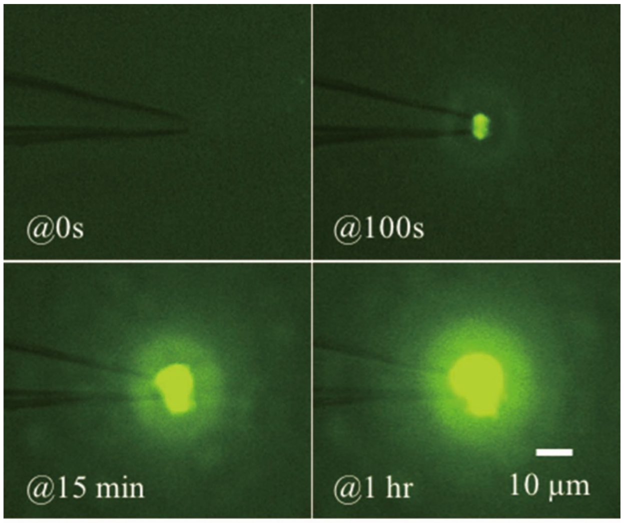

Figure S6. The entrapment results of 510 nm fluorescent COOH-PS beads suspended in 10 mM KCl pH 7.0 using a 2 μm pipette under 10V/cm at different time intervals.

Table S4. Zeta potential of 510 nm COOH-PS beads suspended in different mediums

Suspending medium	Zeta potential (mV)
1 mM KCl	-48.25±1.48
10 mM KCl	-39.13±4.12
100 mM KCl	-21.05±3.79
500 mM KCl	-13.06±2.26
1×PBS	-5.71±0.27

Table S5. Parameters for fabrication of borosilicate nanopipette with different diameters

Diameter of the Pore	FILAMENT	HEAT	PULL	VEL	DELAY
500 nm	1st line: 4<br>2nd line: 4<br>3rd line: 4	1st line: 350<br>2nd line: 350<br>3rd line: 450	1st line: 0<br>2nd line: 0<br>3rd line: 0	1st line: 40<br>2nd line: 40<br>3rd line:60	1st line: 200<br>2nd line: 200<br>3rd line: 200
1000 nm	1st line: 4	1st line: 350	1st line: 0	1st line: 30	1st line: 200
2000 nm	1st line: 4	1st line: 350	1st line: 0	1st line: 24	1st line: 0

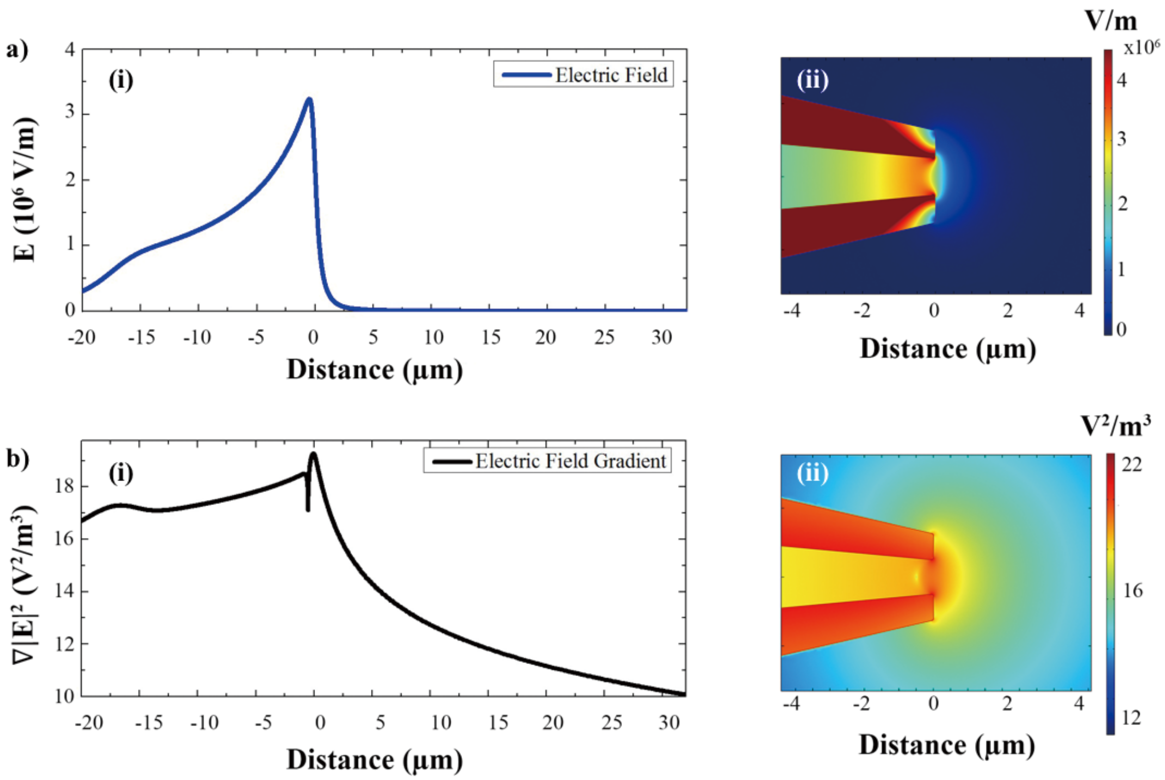

Figure S7. a) (i) The line graph of electric field distribution along the central axis of the pore and (ii) the 2D surface plot of electric field distribution near the tip of the pipette. b) (i)The line graph of electric field gradient distribution along the central axis of the pore and (ii) the 2D surface plot of electric field gradient near the tip of the pipette. The diameter of the pore was 1 μm and 10 V/cm was applied.

Supplemental Videos:

Video S1: 510 nm fluorescently-tagged COOH-PS beads entrapment in 10 mM KCl (pH 7.0)

Video recording of 510 nm COOH-PS beads entrapment in 10 mM KCl (pH 7.0) with a 1 μm pipette and 10 V/cm voltage. The video starts at the 120 second mark after the negative 10 V/cm potential was applied at the base and the polarity was reversed at the 130 second mark. The clip shows that only few beads were trapped with the negative potential at the base, and the trapping results were much more significant after the polarity was reversed to positive potential at the base.

Video S2: Selective entrapment of liposomes in PBS solution

Video recording of the selective entrapment of 100 nm fluorescently-tagged liposomes from 510 nm non-flourecent COOH-PS beads in PBS solution using a 1 µm pipette and 10 V/cm applied E-field. The video starts at the 105 second mark after the negative 10 V/cm potential was applied at the base and the polarity was reversed at the 125 second mark. The clip shows the liposomes were selectively trapped with the negative potential applied at the base and after voltage polarity reversal the non-fluorescent COOH-PS beads were trapped at the tip while the fluorescent liposomes were released.

Video S3: Entrapment of exosomes in PBS solution

Video recording of exosomes entrapment in PBS solution with a 1 µm pipette and 10 V/cm applied voltage. The video starts at the 110 second mark after the positive 10 V/cm potential was applied at the base and the polarity was reversed at the 120 second mark. The clip shows only few exosomes were trapped with the positive bias at the base and more significant trapping was observed after the voltage polarity was reversed to negative bias at the base.
